# Analysis of miRNA expression associated with the *Lr46* gene responsible for APR resistance in wheat (*Triticum aestivum* L.)

**DOI:** 10.1007/s13353-020-00573-5

**Published:** 2020-08-18

**Authors:** Agnieszka Tomkowiak, Tomasz Jędrzejewski, Julia Spychała, Jakub Kuczyński, Michał T. Kwiatek, Agata Tyczewska, Roksana Skowrońska, Tomasz Twardowski

**Affiliations:** 1 Department of Genetics and Plant Breeding, Poznań University of Life Sciences, Dojazd str. 11, 60-632 Poznań, Poland; 2 Institute of Bioorganic Chemistry of the Polish Academy of Sciences, Noskowskiego str. 12/14, 61-704 Poznań, Poland

**Keywords:** Leaf rust, *Lr46* gene, miR164, *Puccinia triticina*, microRNA, Resistance, Wheat

## Abstract

*Lr46/Yr29/Pm39* (*Lr46*) is a gene for slow rusting resistance in wheat. The aim of the study was to analyze the miRNA expression in selected common wheat cultivars carrying resistance genes, *Lr46* among others (HN Rod, Pavon‘S’, Myna‘S’, Frontana‘S’, and Sparrow’S’) in response to leaf rust infection caused by *Puccinia triticina* Erikss. In the Pavon ‘S’, Myna ‘S’, Frontana‘S’, and Sparow‘S’ varieties a product with a length of 242 bp has been identified, which is specific to the *Xwmc44* marker linked to the brown rust resistance gene *Lr46*. In the next step, the differences in the expression of microRNA (miR5085 and miR164) associated with the *Lr46* gene, which is responsible for different resistance of selected wheat cultivars to leaf rust, were examined using emulsion PCR (ddPCR). In the experiment, biotic stress was induced in mature plants by infecting them with fungal spores under controlled conditions in a growth chamber. For analysis the plant material was collected before inoculation and 6, 12, 24, and 48 h after inoculation. The experiments also showed that plant infection with *Puccinia triticina* resulted in an increase in miR164 expression in cultivars carrying the *Lr46* gene. The expression of miR164 remained stable in a control cultivar (HN ROD) lacking this gene. This has proved that miR164 can be involved in leaf rust resistance mechanisms.

## Introduction

The main purpose of breeding programs is to create high-yielding varieties that will also be characterized by high resistance to pathogens. Maximalization of yielding possibilities is often correlated with resistance to fungal pathogens (Strzębicka, 2013). Chemical protection is efficient but expensive way to reduce the fungal infection. Moreover, it is also discouraged due to health risk. Consequently, there is an increasing need to develop new varieties using effective resistance genes, which provide cost-effective and sustainable plant protection. In addition, the use of plant protection products increases production costs and variable and unstable weather conditions often prevent plants spraying being carried out at the optimum time (Tomkowiak et al., 2019). In view of the above, it is strived for well-yielding varieties to have genetically determined disease resistance (Pietrusińska, 2010).

Leaf rust caused by *Puccinia triticina* Erikss is currently considered one of the most dangerous fungal diseases causing high crop losses by reducing the number of kernels per head and the size of the kernels and by lowering test weight and the protein content of the grain. Damage to wheat depends on the growth stage at the time of infection and the overall level of rust severity. High levels of disease before or during flowering usually have the greatest impact on yield (Mehta 2014). Enrichment of wheat gene pools with effective resistance genes, among others to leaf rust, could be a solution to the problem.

It has been reported that the major complexes for long-term immunity are the *Lr46/Yr29/Pm38*, *Lr34/Yr18/Pm38*, and *Sr2/Yr30* gene complexes (Agarwal and Saini, 2009; Kolmer et al. 2008). *Lr46* is one of the genes that provide partial resistance to multiple pathogen species, characterized by a reduced rate of disease development despite an otherwise susceptible infection type. It protects the plant simultaneously against many breeds of pathogens; hence, it is characterized by greater stability under production conditions (Van Ginkel and Rajaram 1993; Krattinger et al. 2009). These genes are expressed, with few exceptions, only at this stage of the plant’s development (Ellis et al. 2014) and a characteristic feature of their action is the slowing down of the pathogen infection process (slow rusting—in the case of rust) and lowering the level of infection. These genes play a special role during the strong pressure of pathogens causing epidemics (Singh and Huerta-Espino, 1997). *Lr46/Yr29/Pm38* gene complex conditioning horizontal immunity functioned worldwide in wheat resistance breeding programs as a source of resistance to brown, yellow, and powdery mildew rust (William et al., 2003; Lillemo et al., 2008; Singh et al., 2013). The *Lr46* gene has been identified in the “Pavon 76” variety on chromosome 1B (Singh et al. 1998). It has long been claimed that this gene is also found in the Americano 25e wheat variety, originating in Uruguay at the beginning of the twentieth century (Kohli, 1986), which was used as a parent in wheat breeding programs in Uruguay and Argentina in the 1920s (Kolmer, 2015). High Indian New Pusa 876 and Sujata varieties were also believed to carry yellow and brown rust resistance genes *Yr29/Lr46* in the 1960s and 1980s (Lan et al., 2015; Ponce-Molina et al., 2018). However, it is still unknown whether the described resistance to many pathogens is the result of pleiotropic effects of a single gene or many cumulative genes (Lagudah).

The *Lr34* gene encoding the ATP binding transporter (ABC transporter—ATP Binding Cassette transporter) (Krattinger et al. 2009) is the best known horizontal type resistance gene. Until now, it has been recognized as a source of persistent and racially nonspecific resistance to brown, yellow, leaf rust, and powdery mildew (*Lr34/Yr18 / Sr57/Pm38*). The *Lr34* gene is common in all hexaploid wheat varieties and is present in several allelic variants. A characteristic feature of this allele is an increase in wheat resistance to infection regardless of race and type of pathogen. The *Lr34* gene is present in European varieties, which have in their pedigree the Mentana and Ardito varieties registered at the beginning of the twentieth century (Lagudah et al. 2009).

It is known, based on the available literature, that the *Lr46* gene provided resistance in wheat varieties infected with various pathogen races originating in Australia (Lagudah et al. 2009). However, little information is available on the roles of these genes during the infection caused by pathogen races that infest crops in Europe. To date, the *Lr46* gene sequence has not been published, although very intensive work is in this field has been conducted, and several candidate genes that provide resistance to mature wheat plants have been identified (Lagudah et al. 2018). In this study the expression profiles of microRNA associated with *Lr46* candidate gene ID CS1B01G454600 proposed by Lagudah et al. (2018) has been studied in 5 wheat cultivars.

With the development of modern molecular techniques, it has been noticed that miRNAs play an important role in regulating gene expression. The basic mechanism of miRNA expression is now known and how it affects mRNA. In most cases, RNA polymerase II is responsible for miRNA transcription. At this stage, pri-miRNA is formed (composed of several thousand base pairs). The Drosha enzyme, with RNase III activity, cuts double-stranded pri-miRNA, resulting in shorter pre-miRNA. Transport to the cytoplasm of miRNA precursors is carried out using Exportin-5 and its RAN-GTP cofactor. There, Dicer—another enzyme with RNase III activity—cuts the pre-miRNA, resulting in 18–22 bp miRNA formation (Tyczewska et al. 2016). The miRNA then binds to the RISC protein complex, the active complex contains in most cases is the antisense strand of the miRNA (the second strand degrades). The mechanism of action of the RISC-miRNA complex on gene expression is based on recognition of the target transcript by miRNA, based on the complementarity of the sequences of both types of RNA (Chu et al. 2016). If the homology of the miRNA sequence and target mRNA is high, then the Argonaute2 protein of the RISC complex cuts the mRNA and/or degrades the messenger RNA molecule. Most often, the RISC complex binds to the transcript 3'UTR region, thereby causing repression of mRNA translation. In this case, the homology between miRNA and information RNA is only partial (Liu et al. 2014).

## Material and methods

### Plant material

Plant material used for the study consisted of common 5 wheat cultivars named Pavon‘S’, Myna‘S’, Frontana‘S’, Sparrow‘S’, and HN ROD; the cultivars were derived from the National Small Grain Collection located at the Agricultural Research Station in Aberdeen, WA, USA. Four of the analyzed cultivars have the leaf rust resistance gene *Lr46* (Pavon‘S’, Myna‘S’, Frontana‘S’, and Sparrow‘S’), while the HN ROD, which does not carry this gene, served as a negative control.

### Methods

#### Experiment in controlled conditions, established in a growth chamber

For each cultivar, 5 plants were planted into pots, in two variants (inoculated and non-inoculated), the experiment was done in triplicate; hence, there were 30 pots in total (5 cultivars × 3 replicates × 2 variants). Leaf tissue fragments for molecular analysis were collected from each pot before inoculation and 6, 12, 24, and 48 h after inoculation. In total, 150 samples (5 cultivars × 3 biological replicates × 5 time points (0, 6, 12, 24 and 48 h) × 2 variants (inoculated and non-inoculated)) were collected for expression analysis of miRNA associated with the *Lr46* gene. On April 24, 2019, wheat leaves infected with brown rust were taken from a field belonging to the Poznań Plant Breeding in Antoniny (N: 51° 30′ 44″–E: 17° 50′ 51″). The leaves that had a large amount of urediniospores were selected. In the laboratory, the spores were washed out with sterile water. Infectious material was a mixture of 4 *Puccinia triticina* isolates. Seven-week-old wheat plants were inoculated by spraying whole plants with a spore suspension at a concentration of about 5 × 10^5^ spores/ml.

#### Identification of Xwmc44 genetic marker of the Lr46 gene

To confirm the presence of a *Xwmc44* genetic marker in tested wheat cultivars genomic DNA was isolated from leaf tissues using the GeneMATRIX kit (EURx Ltd., Poland), according to the attached procedure. The concentration and quality of DNA were checked using a DeNovix spectrophotometer at 260 nm. Molecular microsatellite (SSR) marker *Xwmc44*, which is coupled to *Lr46* gene, was used to prove the gene’s presence in tested wheat lines. *Xwmc44* marker locus is located 0.4 cM from quantitative trait loci (QTL) for *Lr46*. The presence of the product of 242-bp indicates the presence of the *Lr46* gene in the genomes. The sequences of primers used in the polymerase chain reaction (PCR) are *WMC44*F 5′-GGTCTTCTGGGCTTTGATCCTG-3′, *WMC44*R 5′-GTTGCTAGGGACCCGTAGTGG-3′ (Suenaga et al. 2003).

The PCR reaction mixture (12.75 μl) contained the following reagents: water—5 μl, DreamTaq™ Green PCR Master Mix (ThermoFisher Scientific)—6.26 μl, primers—2 × 0.25 μl (final concentration - 20 μM), and DNA template—1 μl. The conditions for PCR reaction were as follows: pre-denaturation—5 min at 94 °C, 40 cycles of denaturation—45 s at 94 °C, primer annealing—1 min at 54 °C, elongation—1 min at 72 °C, and the final elongation—5 min at 72 °C, storage at 4 °C for max 24 h. The reaction was carried out in Labcycler thermal cyclers (SensoQuest GmbH). Following the amplification, 0.5 μl Midori Green Direct (NIPPON Genetics EUROPE) was added to the products and the mixtures were separated on 2% agarose (SIGMA) gel in 1× TBE buffer (BioShop) at 100 V for 2.5 h. A Molecular Imager Gel Doc™ XR UV system was used with the Biorad Bio Image™ Software to visualize the PCR products.

#### Expression analysis of selected miRNAs associated with the Lr46 gene

In the first stage, the sequence of *Lr46* candidate gene (ID CS1B01G454600), proposed by Lagudah et al. (2018), was searched and compared in the Ensembl plants and NCBI database. Based on the sequence of this gene in the psRNAtarget database (http://plantgrn.noble.org/psRNATarget/), two microRNAs that are complementary to the candidate gene sequence were selected for tests: miR5085 and miR164. The sequences of selected miRNAs were obtained from miRBase, and reverse transcription and ddPCR primers were designed using IDT (Integrated DNA Technologies).

A mirVana™ miRNA Isolation Kit, with phenol (ThermoFisher Scientific), was used to isolate the RNA fraction containing microRNAs. Isolation was performed according to the procedure provided by the manufacturer. The concentration and quality of isolated RNA were checked using a DeNovix spectrophotometer at 260 nm. A set amount of extracted RNA (1 μg sRNA and 1.5 μg of total RNA) was reverse-transcribed using SuperScript IV Reverse Transcriptase (ThermoFisher), stem-loop primers, and the following protocol: incubation for 30 min at 16 °C; 60 cycles at 30 °C for 30 s, 42 °C for 30 s, and 50 °C for 1 s; and incubation at 85 °C for 5 min. To quantify the number of miRNA molecules in the plant samples, a ddPCR mixture composed of 10 μl of ddPCR Super Mix Eva Green, primers (the final concentration of each primer was 200 nM), template (reverse-transcribed, elongated miRNA), and RNase-free H_2_O was used. A 20-μl reaction mixture was used to generate the droplets in an 8-well cartridge using a QX100 droplet generator (Bio-Rad). The droplets were carefully transferred to a 96-well ddPCR plate and heat-sealed with foil (Bio-Rad). The cDNA was then amplified in a T100 PCR thermal cycler (Bio-Rad) under the following cycling conditions: 5 min of denaturation at 95 °C, followed by 40 cycles with a three-step thermal profile of 30 s of denaturation at 95 °C, 30 s of annealing at 58 °C, and 45 s of extension at 72 °C. After that, the products were kept at 72 °C for 2 min for the final extension. After amplification, the products were cooled to 4 °C for 5 min and then heated to 90 °C for 5 min and finally cooled again to 12 °C. The droplets were quantified in a QX100 droplet reader (Bio-Rad). Data acquisition and analysis were performed using QuantaSoft software (Bio-Rad). The positive droplets containing the amplification products were discriminated from the negative droplets by setting the fluorescence amplitude threshold to the lowest value of the positive droplet cluster.

## Results

**Fig. 1:**
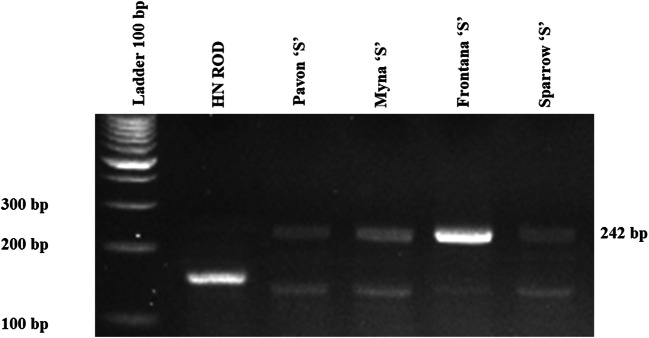
Electropherogram showing the presence/absence of a band amplified from*Xwmc44* marker (242 bp) in common wheat cultivars *Triticum aestivum* ssp. *vulgare* L

As expected, 4 out of 5 tested wheat lines (Pavon‘S’, Myna‘S’, Frontana‘S’, and Sparrow‘S’) carried the *Lr46* gene. The results of analyses performed to detect *Xwmc44* genetic marker, that is linked to the *Lr46* leaf rust resistance gene, identified the specific product of 242 bp. For the 4 cultivars that tested positive for the *Lr46* gene, non-specific product of 140 bp was also observed. *Xwmc44* marker was not detected in the control cultivar HN ROD, confirming it did not have the *Lr46* gene (Fig. 1). In the performed experiments the expression of microRNA associated with the *Lr46* candidate gene (ID CS1B01G454600) was proposed by Lagudah et al. (2018), which were identified based on bioinformatic search that has been analyzed. Based on the sequence of the gene of interest two microRNAs complementary to the candidate gene sequence (from the psRNAtarget database (http://plantgrn.noble.org/psRNA Target/) were selected: miR5085 and miR164. The expression levels of both miRNAs were tested using the ddPCR method. Despite many replicates, the results of analyses concerning miR5085 did not allow to obtain sufficient number of copies to be able to correctly infer its expression levels.

In turn, the experiments on miR164 allowed to fully analyze the changes in its expression in individual cultivars, resulting from plant infection by *Puccinia triticina*. In the non-inoculated experimental variant, miR164 concentrations in the analyzed cultivars in all replicates (biological and technical) and time points (0, 6, 12, 24, 48 h) did not show significant differences; i.e., no miR164 expression was observed. On the other hand, the expression of miR164 was observed in all the inoculated variants. In most cases no significant differences were recorded between technical and biological repetitions, which were the result of correctly optimized reaction conditions. The average results from biological and technical replicates regarding miR164 expression levels in tested wheat cultivars are presented in Table 1.

**Table 1:** The concentration of miR164 in the analyzed cultivars (tested before inoculation and after 6, 12, 24, and 48 h after inoculation in biological and technical replicates)—average of replicates

Analyzed genotypes	miR164 concentration (copies/μl)—average of replicates				
	Before inoculation	6 h after inoculation	12 h after inoculation	24 h after inoculation	48 h after inoculation
HN ROD	48.1	26.8	36	46	39
Pavon‘S’	193.8	15.8	60.6	116.3	115.1
Myna‘S’	122.8	17.5	42.8	68.3	224.1
Frontana‘S’	110.3	70.1	91.6	103.8	181.1
Sparrow‘S’	134.1	45.6	54.1	295.8	45.3

**Fig. 2:**
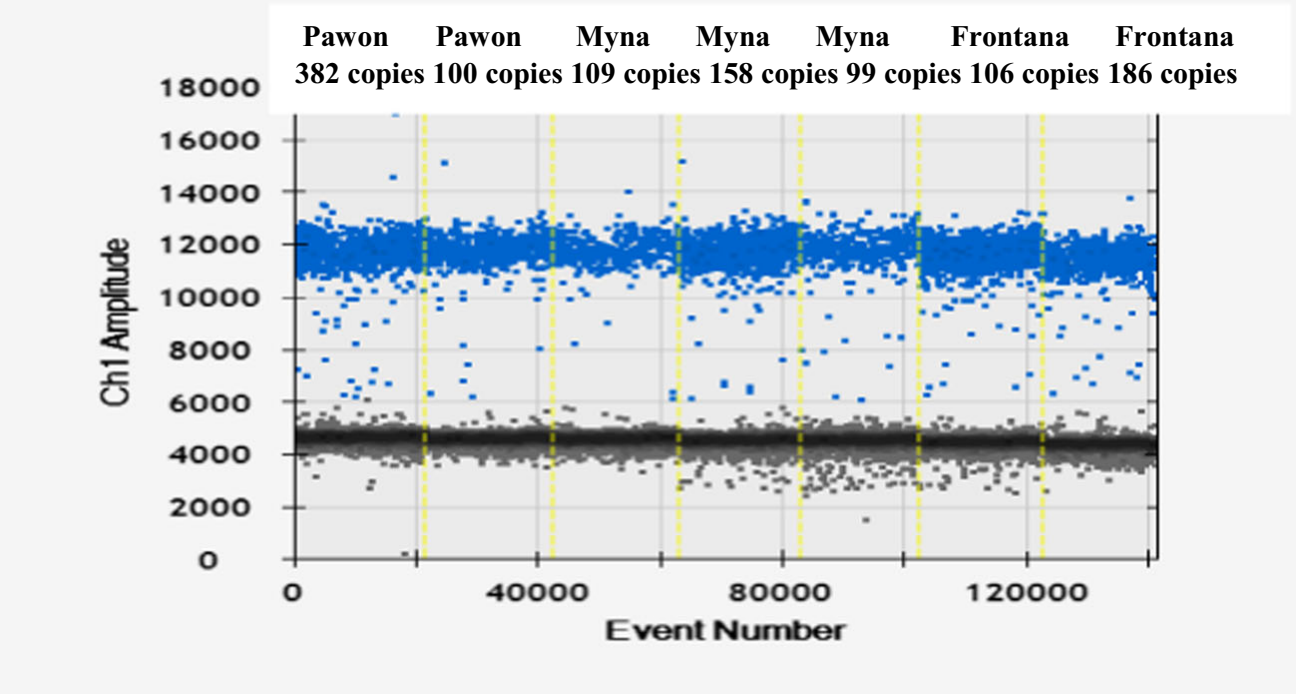
Sample ddPCR reaction showing miR164 concentration (number of copies/μl) for selected cultivars and replicates

**Fig. 3:**
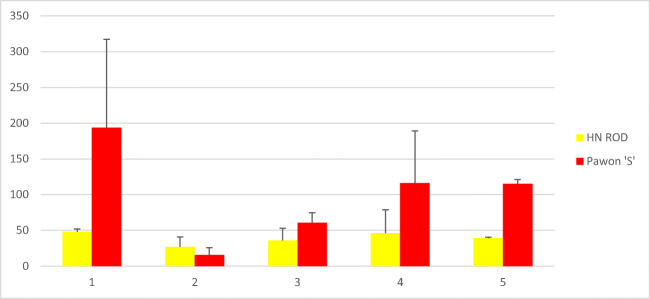
Graph showing miR164 concentration (number of copies/μl) of the cultivar Pavon ‘S’ compared to the cultivar HN ROD before and 6, 12, 24, and 48 h after inoculation with *Puccinia triticina*

**Fig. 4:**
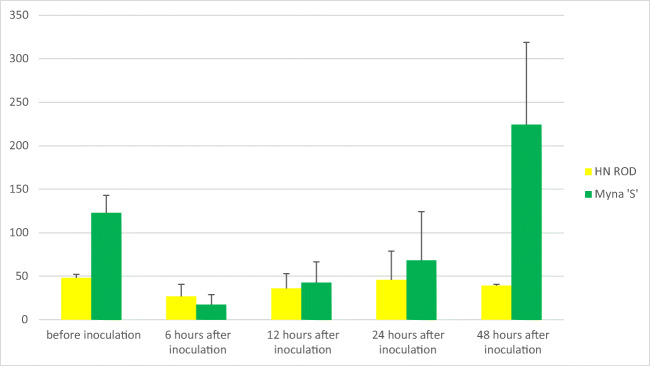
Graph showing miR164 concentration (number of copies/μl) of the cultivar Myna‘S’ compared to the cultivar HN ROD before and 6, 12, 24, and 48 h after inoculation inoculation with *Puccinia triticina*

**Fig. 5:**
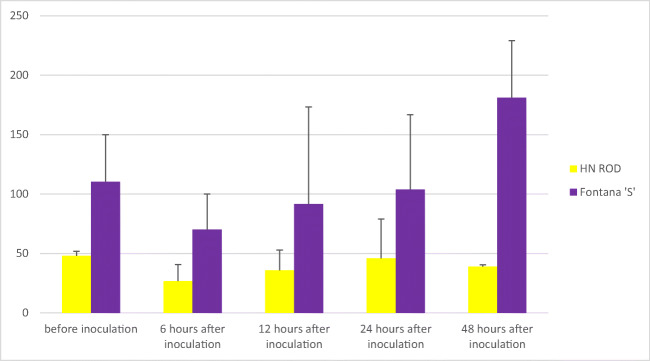
Graph showing miR164 concentration (number of copies/μl) of the cultivar Frontana‘S’ compared to the cultivar HN ROD before and 6, 12, 24, and 48 h after inoculation inoculation with *Puccinia triticina*

**Fig. 6:**
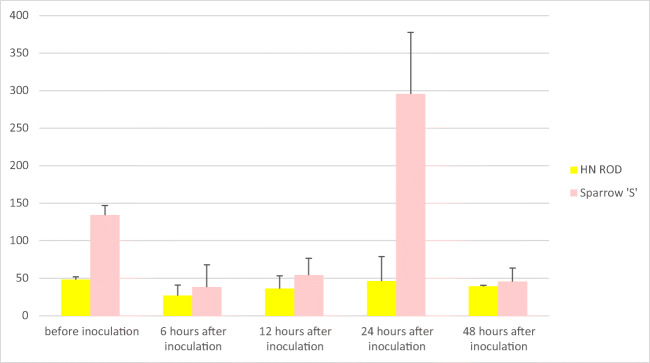
Graph showing miR164 concentration (number of copies/μl) of the cultivar Sparrow‘S’ compared to the cultivar HN ROD before and 6, 12, 24, and 48 h after inoculation inoculation with *Puccinia triticina*

Importantly all cultivars except the control one (HN ROD) responded to *Puccinia triticina* infection. A sharp decrease in miR164 expression level was observed in all cultivars possessing the *Lr46* gene (Pavon‘S’, Myna‘S’, Frontana‘S’, and Sparrow‘S’) 6 h after inoculation (Table 1). The strongest reaction to stress was noted for Pavon‘S’ and Myna‘S’ cultivars, as their miR164 copy number was reduced from 193.8 to 15.3 copies/μl and from 122.8 to 17.5 copies/μl, respectively. Moreover, in time (12 h, 24 h, and 48 h after inoculation), a gradual increase in miR164 level was observed in the Pavon‘S’, Myna‘S’, and Frontana‘S’ cultivars (Table 1). Similar to other cultivars, an increase in miR164 was observed in the cultivar Sparrow’S’ 12 h and 24 h after inoculation, and then the copy number of miR164 began to decrease and after 48 h reached 45.3 copies/μl. Thus, a decrease in miR164 concentration after 6 h could be observed in all analyzed variants with the *Lr46* gene after inoculation-induced stress, followed by an increase over time. The highest miR164 expression level was observed for Myna‘S’ and Frontana‘S’ (Table 1) as the number of miRNA copies per microliter in both cultivars after 48 h exceeded the initial expression level recorded before inoculation (Myna‘S’—122.8 copies/μl before inoculation, 224.1 copies/μl after inoculation; Frontana‘S’—110.3 copies/μl before inoculation, 181.1 copies/μl after inoculation). In case of the control cultivar HN ROD, miR164 level in biological and technical repeats (regardless of time point) was at a similar level. The observed slight differences could be due to ddPCR reaction conditions. An example of ddPCR analysis is shown in Fig. 2. The expression levels of miR164 in 4 cultivars carrying *Lr46* gene in comparison to the control cultivar HN ROD are shown in separate graphs (Figs. 3, 4, 5, and 6). The attached graphs clearly show that in contrast to other cultivars carrying the *Lr46* gene, we did not observe clear differences in miRNA expression in the control cultivar HN ROD after plant infection with *Puccinia triticina*. As mentioned above, the largest increase in miR164 expression occurred in cultivars Myna‘S’ and Frontana‘S’ (Figs. 4 and 5).

## Discussion

Wheat vegetation has two periods when it is most susceptible to infection. The first period is the beginning of the shooting stage, because then wheat plants are at risk of blade fragility. In turn, the beginning of heading is the time when wheat is threatened by septoria leaf blotch, leaf rust, and powdery mildew of cereals and grasses. Depending on the agrotechnical and habitat conditions, these diseases can cause a decrease in yield even up to 19% (Pietrusińska, 2010). The pathogen *Puccinia triticina* Erikss causes leaf rust, the symptoms of which are visible on leaf blades, leaf sheaths, and stalks. In Poland, it is the most common rust in cereal crops (Strzembicka et al., 2013). The reason for the decrease in yield is the low-grade grain from which the flour has a lower baking value. Straw of worse quality is also obtained as a result of plant infestation. The most effective method of plant protection so far is the cultivation of resistant cultivars.

Wheat gene conditioning resistance to pathogens, insects, viruses, and nematodes are divided into 25 classes and described in the Catalogue of Gene Symbols for Wheat. Genes that determine resistance to leaf rust constitute the largest group, and they have been assigned the symbol *Lr* (Leaf rust) in the generally accepted nomenclature. Most *Lr* genes are Major Resistance Genes (Ellis, 2014), which determine monogenic resistance, according to Flor’s “gene-to-gene” theory (Gogół et al., 2015). Some genes may condition plant resistance at the seedling plant resistance (SPR) stage (Spanic et al. 2014). Others, in turn, determine adult plant resistance (APR) and these include, among others, the *Lr34* and *Lr46* genes (Suenaga et al., 2003). APR is a very important type of resistance because most frequently it is racially non-specific, more persistent, and reduces the risk of epidemics (Lagudah et al. 2009).

In the current study, the 242-bp product of amplification of *Xwmc44* marker, which is linked to the leaf rust resistance gene *Lr46*, was identified in 4 out of 5 analyzed cultivars (Pavon‘S’, Myna‘S’, Frontana‘S’, and Sparrow‘S’) confirming the presence of the desired gene. Cobo et al. (2018) mapped the *Yr29* yellow rust resistance locus in the 332-kb region of the 1BL chromosome arm in adult plants. This region is rich in genes that are also associated with resistance to brown rust *Lr46*, stem rust *Sr58*, and powdery mildew *Pm39*. The authors marked this place on the chromosome as *QYr.ucw-1BL*. Resistance genes present in this chromosome region have been effective for over 60 years and are widespread in CIMMYT wheat germplasm (Singh et al., 2011). Cobo et al. (2018) reported that single genes found at this locus provide resistance to many different pathogens (Krattinger et al., 2009; Moore et al., 2015). However, the authors do not rule out the hypothesis that these genes may be combined into one mechanism of immunity (Lagudah, 2011). Cobo et al. (2018) showed that the 332-kb *csLV46G22* marker flanking the QYr.ucw-1BL candidate region has been mapped and is closely related to *Yr29* (Ren et al. 2017; Dong et al. 2017; Ponce-Molina et al., 2018). The latest maps for *Yr29*/*Lr46* from Pavon 76 place this locus between TraesCS1B01G453900 and *csLV46G22* (Lagudah, unpublished data, 2018), a region very similar to the candidate region of the 332 kb gene for *QYr.ucw-1BL* identified here. The *Yr29* and *QYr.ucw-1BL* candidate gene regions differ only in the exclusion of T*raesCS1B01G453700* from the *Yr29* region and its partial incorporation into the 332-kb *QYr.ucw-1BL* region. Therefore, this work analyzed resistance mechanisms of the *Lr46* gene in response to plant infection by *Puccinia triticina*.

The *Lr46* candidate gene has been selected based on a work conducted by Lagudah et al. (2018), and the miRNAs for tests were chosen based on sequence complementarity to the gene of interest. Out of two identified miRNAs, it was impossible to detect and analyze the expression profile of miR5085. The present study also showed that plant infection with *Puccinia triticina* resulted in changes in miR164 expression levels in cultivars carrying the *Lr46* gene (Pavon‘S’, Myna‘S’, Frontana‘S’, and Sparrow‘S’). Directly after inoculation, within 6 h, the miR164 level dropped, which in consequence may lead to an increase in the *Lr46* gene level. This is important in the first stages of infection when a quick response from the resistance gene is required. It has been shown that as the infection progressed, the level of miR164 increased gradually and the level of Lr46 expression decreased. Thus, it can be stated that miR164 is involved in the mechanisms of plant resistance to leaf rust. The expression of miR164 remained stable in the control cultivar (HN ROD) lacking this gene.

Plants exposed to stress condtions use many gene regulation mechanisms, including post-transcriptional regulation, to restore cellular homeostasis. To date, many authors reported that miRNAs are involved in defending plants against pathogens. Wang et al. (2017) claimed that the regulation of GhMKK6 transcription via ghr-miR5272a contributed to the cotton plant’s immune response. Among genes that are post-transcriptionally regulated by miRNA, thus repressing/promoting the expression of downstream genes, many are transcriptional factors, including NAC, MYB, and WRKY (Hao et al., 2012; Yu et al., 2012; Hu et al. 2020). These transcriptional factors influence the immune response to both abiotic and biotic stress conditions by controlling numerous aspects of development, such as cellular proliferation and differentiation, metabolism of phenylpropanoids, and hormone signaling (Ambawat et al. 2013). The name NAC comes from the acronym: NAM (no apical meristem), ATAF (*Arabidopsis* transcription activation factor), and CUC (cup-shaped cotyledon), and the mRNA encoded by NAC contains miR164 complementary site, with several mismatches (Hu et al. 2020). Previously, it has been shown that NAC is negatively regulated by miR164 in *Arabidopsis* (Sieber et al., 2007). Additionally, Kim et al. (2009) reported that the NAC transcription factor from *Arabidopsis* was involved in leaf cell death via the regulation by miRNA164. The function of miR164 in plant responses to biotic stresses has been verified through the regulation of its corresponding target genes in *Populus trichocarpa* and wheat (Zhao et al., 2012; Feng et al., 2014). Among these target genes, TaNAC21/22 participated in wheat plant resistance to stripe rust regulated by tae-miR164 (Feng et al., 2014). Our results show that miR164 likely has a role in managing the expression of the leaf rust resistance gene *Lr46* in wheat cultivars (Pavon‘S’, Myna‘S’, Frontana‘S’, and Sparrow‘S’).

Hormonal signaling in plants is a key mechanism that functions during stress responses. Studies conducted on *Arabidopsis* pointed to the role of five conserved miRNAs, i.e., miR159, miR164, miR167, miR171, and miR444 in hormonal signaling (Kumar et al., 2014). Moreover, research conducted by Kumar et al. (2016) allowed to detect leaf rust responsive miRNAs in wheat by comparing control and pathogen-inoculated near-isogenic lines (NILs) of HD2329 with (resistant) and without (susceptible) *Lr24* gene. Analysis confirmed the positive contribution of five miRNAs in response to *P. recondita* infection: miR167, miR168, miR398, miR399, and miR156. The pathogen-inoculated resistant plants had higher miRNA levels compared with the pathogen infected susceptible plants (Kumar et al., 2014), which is in parallel to our study. Additionally, miRNAs can also affect wheat response to powdery mildew infection. Some of the tested miRNAs showed different expression patterns in response to powdery mildew infection: miR156, miR159, miR164, miR171, and miR396 had decreased expression, and miR393, miR444, and miR827 increased (Khraiwesh et al., 2012). As follows from the studies of Li et al. (2017), miR9773 plays an important role in the response to wheat biotic stress.

## Conclusions

APR genes are successfully used in breeding programs CIMMYT (The International Maize and Wheat Improvement Center). These genes have been proven to slow down a pathogen infection. This information is confirmed by the presented research because immediately after inoculation, an intense decrease in miR164 level was observed. This miRNA targets *Lr46* candidate gene. The decrease in miR164 may be associated with activation of the *Lr46* gene-dependent resistance mechanisms, observed as a rapid gene response to the pathogen. The stronger the pathogenic pressure was, the longer it took an increase in the amount of miR164 and at the same time weakened resistance mechanisms were observed. On this basis, it can be concluded that the *Lr46* gene actively participates in the plant’s resistance response to *Puccinia triticina* infection.

## Data Availability

Not applicable.
